# Meaning in Life Mediates Between Emotional Deregulation and Eating Disorders Psychopathology: A Research From the Meaning-Making Model of Eating Disorders

**DOI:** 10.3389/fpsyg.2021.635742

**Published:** 2021-03-23

**Authors:** Jose H. Marco, Montserrat Cañabate, Cristina Martinez, Rosa M. Baños, Verónica Guillen, Sandra Perez

**Affiliations:** ^1^Personality, Assessment and Psychological Treatment, University of Valencia, Valencia, Spain; ^2^University CEU Cardenal Herrera, Castellón de la Plana, Spain; ^3^Hospital Clínico Universitario de Valencia, Valencia, Spain; ^4^Personality, Assessment and Treatments, Catholic University of Valencia San Vicente Martyr, Valencia, Spain; ^5^CIBER Fisiopatología Obesidad y Nutrición (CIBEROBN), Madrid, Spain

**Keywords:** meaning in life, emotional deregulation, obesity, young women, eating disorders, meaning-making model

## Abstract

Emotional dysregulation, age, gender, and obesity are transdiagnostic risk factors for the development and maintenance of eating disorders (EDs). Previous studies found that patients with ED had less meaning in life than the non-clinical population, and that meaning in life acted as a buffer in the course of ED; however, to the data, there are no studies about the mediator role of meaning in life in association between the emotional dysregulation and the ED psychopathology.

**Objective:** To analyze the mediating role of meaning in life in the relationship between emotional dysregulation and the ED psychopathology in three samples with diverse risk factors for ED.

**Method:** Sample 1, *n* = 153 undergraduate young women; sample 2, *n* = 122 participants with obesity; and sample 3, *n* = 292 participants with ED. Multiple mediation analysis was performed.

**Results:** Sample 1: meaning in life showed a mediation effect between emotional dysregulation and the ED psychopathology (direct effect β = 0.390, *p* < 0.05) (indirect effect β = 0.227, *p* < 0.05), body satisfaction (direct effect β = −0.017, *p* < 0.05) (indirect effect β = −0.013, *p* < 0.01), and depression symptoms (direct effect β = 1.112, *p* < 0.001) (indirect effect β = 0.414, *p* < 0.001); sample 2: meaning in life showed a mediation effect between emotional dysregulation and binge eating and purging behaviors (direct effect β = 0.194, *p* < 0.01) (indirect effect β = 0.054, *p* < 0.05) and depression symptoms (direct effect β = 0.357, *p* < 0.001) (indirect effect β = 0.063, *p* < 0.05); sample 3: meaning in life showed a mediation effect between emotional dysregulation and the ED psychopathology (direct effect β = 0.884, *p* < 0.001) (indirect effect β = 0.252, *p* < 0.007), body satisfaction (direct effect β = −0.033, *p* < 0.05) (indirect effect β = −0.021, *p* < 0.001), borderline symptoms (direct effect β = 0.040, *p* < 0.001) (indirect effect β = 0.025, *p* < 0.001), and hopelessness (direct effect β = 0.211, *p* < 0.001) (indirect effect β = 0.087, *p* < 0.001).

**Conclusions:** These studies suggest the importance of considering meaning in life as a variable in the onset and maintenance of ED.

## Introduction

Although the etiology of eating disorders (EDs) is multi-factorial (Long et al., 2017), there is a broad consensus in the literature that emotional dysregulation (Monell et al., 2015; Mallorquí-Bagué et al., 2018), age (adolescents and young adults), gender (female) (Rosenvinge and Pettersen, 2015a), and a personal history of obesity are transdiagnostic risk factors in the development and maintenance of ED (Micanti et al., 2017).

Emotional regulation can be defined as the ability to identify and modulate emotions (Gross, 1998), and the level of emotional dysregulation has been found to be associated with the severity of cognitive symptoms in ED (Aldao et al., 2010; Pisetsky et al., 2017) and predict the maintenance of AN after the treatment ends (Racine and Wildes, 2015). Moreover, engaging in the emotional strategy of avoiding negative and positive emotions becomes a maintainer of depressive and anxiety symptoms in participants with ED (Wildes et al., 2010).

Regarding age and gender, EDs affect between 11 and 15% of women, and there is a broad consensus in the literature that being a young woman is a specific risk factor for EDs (Allen et al., 2013). Theories suggest that, in young women, the internalization of the dominant beauty ideals, based on the idealization of thinness, would lead to body dissatisfaction in people with other vulnerability factors, such as overweight, negative affect or depression, perfectionism, and low self-esteem, which would contribute to extreme weight control behaviors and lead them to develop EDs (Rosenvinge and Pettersen, 2015b).

Obesity is another specific risk factor for developing an ED (Guisado et al., 2002), and ~30–80% of individuals with bulimia nervosa (BN), binge eating disorder (BED), or other specified feeding or eating disorders (OSFED) are people with obesity (Villarejo et al., 2012). Some studies have shown that people with obesity share several clinical characteristics with people with ED (Brone and Fisher, 1988), as well as etiological, psychological, and social factors (Jarman et al., 1991), such as rumination, negative attitudes toward food and the tendency to diet (Guisado et al., 2002), and body dissatisfaction (Weinberger et al., 2016). Moreover, participants with obesity have a high comorbidity with depression (Dixon et al., 2003), anxiety disorders, BN, and BED (Black et al., 1992).

Despite the large number of studies on risk factors and prevention in ED, the incidence of EDs has kept stable in mental health services from 1970 to the first decades of the 21st century (Hoek, 2016). This could suggest that there may have been an increase in the incidence of ED not treated in mental health settings. Therefore, it is necessary to examine the preventive factors targeting shared risk of eating-/weight-related issues and explore new paths that can improve the existing prevention programs and the efficacy of the treatments. In this sense, a variable that has been found to be negatively associated with the psychopathology of ED is the meaning in life (e.g., Gongora, 2014; Brassai et al., 2015; Marco et al., 2017).

Meaning in life is made up of three dimensions that are interconnected and interact: (a) Coherence, the degree to which people feel that the world in which they live is an organized, structured, predictable, and explainable whole; (b) Purpose, which refers to the way people experience that their life is oriented and guided by important life goals and values; (c) Significance, which refers to the feeling that life itself has inherent value and involves having a life worth living (Martela and Steger, 2016). There are already studies that found that patients with ED had less meaning in life than the non-clinical population (Marco et al., 2017). Furthermore, meaning in life was highly and positively related to body satisfaction and negatively associated with concern about being overweight and negative attitudes toward food (Marco et al., 2019). Subsequently, a longitudinal study found that meaning in life was a buffer of the course of the ED, specifically of dysfunctional attitudes and behaviors toward food, hopelessness, suicidal ideation, and impulsiveness and emotional instability (Marco et al., 2020b).

Moreover, several studies suggested that meaning in life could be an important variable in the recovery from ED. de Vos et al. (2017) carried out a study to identify relevant criteria for ED recovery from the perspective of recovered patients, and they found that meaning in life and purpose were a main criterion for recovery. Garrett (1997) found that recovery involves escaping the obsession with food and weight, believing that life is meaningful and one is worthwhile, and having the conviction to not return to starvation. Bowlby et al. (2015) found that all ED participants described experiences of creating meaning and purpose in their lives outside of ED as a result of the recovery process.

For a more integrated and operational view of the role of meaning in life in ED patients, Marco et al. (2020b) proposed the Meaning-Making Model in Eating Disorders (MMMED). This model shares Frankl's (2006) premises: (a) the need to find meaning in life is a fundamental motivating factor in human beings; (b) low meaning in life is a vulnerability factor for developing emotional disorders, and meaning in life is a protector factor against psychopathology; (c) meaning in life can only be discovered in genuine and authentic sources of meaning (creating something, loving someone or something, facing a painful and unavoidable situation) that will lead to the discovery of an individual meaning in life at each moment and for each person differently; (d) the search for meaning in dysfunctional sources would impede the development of authentic and genuine meaning, leading to the absence of meaning in life.

The MMMED states that people with high vulnerability to ED (young women with low self-esteem, high perfectionism, high body mass index, emotional dysregulation, and depression) when they face a situation that has led them to have low or no meaning in life, generally produced by an event that violates their global scheme of values, ideals, and goals (for example, an increase in weight would violate the global scheme “I have to control my weight”; “I need to have a good appearance”; “I am perfect”; “I want to be anorexic”). This violation of global meaning would lead to an absence of meaning and to launching a set of processes oriented toward meaning-making. These processes in people with high vulnerability to ED could include controlling food to lose weight, checking behaviors of the body, ruminant thoughts about food and the body, increased negative affect, and emotional dysregulation, which would lead to the patient losing weight in the case of anorexia nervosa (AN) or to developing binge eating and vomiting (in the case of BED or BN). Thus, according to the MMMED, this meaning-making process could have two consequences. In the short term, the ED symptoms could be dysfunctional strategies that give people a sense of structure, consistency in their lives, and identity (Serpell et al., 1999; Fox and Leung, 2009), creating a new coherent meaning in life (“After losing weight I am attractive, I control my body, and so I am perfect”; “I am Anorexic”). However, in the long term, if individuals with ED are oriented toward dysfunctional goals and values, including control of their bodies, weight, and food, and they avoid negative emotions and anxiety situations, which keeps them from developing an authentic and genuine sense of meaning in life, this leads to an absence of meaning in life, depression, hopelessness, suicidal ideation, and developing characteristic symptoms of borderline personality disorder (Marco et al., 2020b).

Regarding emotional deregulation, the MMMED model suggests that when there is an absence of or low meaning in life, the intensity of negative emotions and emotional instability will increase (e.g., negative affect or depression), and emotional regulation strategies (e.g., suppression, elaboration, avoidance, etc.) would either facilitate or prevent meaning-making. In the same way, developing meaning in life could buffer the emotional deregulation (Marco et al., 2017). Thus, the MMMED suggests that emotional regulation strategies will be adaptive or maladaptive depending on whether they lead to meaning-making in this specific situation or event.

The MMMED suggests that meaning in life would also act as a mediator in the relationship between emotional dysregulation and ED psychopathology. If meaning in life is a mediating variable of the risk factors for ED, this mediating role should be found in participants with different levels of risk of having an ED, that is, in participants with low risk (young women without an ED), participants with moderate risk (people with obesity), and, finally, patients diagnosed with ED. To date, the mediator role of meaning in life in several samples with different risk factors for ED has not been analyzed.

Thus, the aim of the present study is to analyze the mediating role of meaning in life in the relationship between emotional dysregulation and the ED psychopathology in three samples with diverse risk factors for ED: participants young women (sample 1), participants with obesity (sample 2), and participants with ED (sample 3). We hypothesize: (a) meaning in life could be a mediator in the association between emotional dysregulation and body dissatisfaction, depression, and ED psychopathology in women under 25 years old; (b) meaning in life could be a mediator in the association between emotional dysregulation and depression, BN, and BED psychopathology in participants with obesity; and (c) meaning in life could be a mediator in the association between emotional dysregulation and body dissatisfaction, ED psychopathology, borderline personality symptoms, and hopelessness in participants with ED.

## Method

### Participants

For the present study, the three samples recruited were selected by the same research team and belong to the same line of research entitled: Is meaning in life an important variable in the psychopathology of ED? To answer this general question, three samples were selected: sample 1 consisted of participants without a diagnosis of ED or obesity, and it was obtained in January 2017 (research code UCV2017–2018/116). Sample 2 consisted of participants with obesity who were recruited from 2016 to 2018 (research code FPNT-CEB-04-2015/0402), and sample 3 consisted of participants with a diagnosis of ED who were recruited in 2015–2018 (research code UCV2013–2014/0023). The general aim of the study was to analyze whether meaning in life is a mediator in the relationship between emotional dysregulation and the psychopathology of ED in groups with a different risk of developing an ED, the non-clinical population (low risk), the population with obesity (moderate risk), and the clinical population (high risk). Table 1 shows the demographic and clinical characteristics of the three samples.

**Table 1:** Demographic and clinical characteristics of the three samples.

	**Sample 1 Non-clinical**	**Sample 2 Participants with obesity**	**Sample 3 Participants with eating disorders**
*N*	156	122	292
Age	21.06 (2.12)	47 (9.89)	24.21 (11.01)
Gender	Women 100% *n* = 156	Women 64.8% *n* = 79	Women 93.5% *n* = 273
Ethnic composition	100% Caucasian	100% Caucasian	100% Caucasian
Marital status			
Single, separated, divorced	60.7%, *n* = 95	39.7%, *n* = 48	58.6%, *n* = 171
Living a couple or married	39.3%, *n*= 58	60.3%, *n* = 74	41.4%, *n* = 121
Mean body mass index	21.72 (3.38)	44.41 (6.03)	22.60 (7.26)
Level of studies			
Had no studies	–	2.45% (*n* = 3)	–
Primary studies	–	43.44% (*n* = 53)	26.4%, *n* = 77
Secondary studies	–	41% (*n* = 50)	50.7%, *n* = 148
Higher studies	100%	13.11% (*n* = 16)	22.9%, *n* = 67
Comorbidity			
Medical diseases	–	77.5% (*n* = 93)	–
Mental disorders	–	9.8% (*n* = 12)	22.6%, *n* = 66
Sample provenance	University	Bariatric Surgery Service of hospital	Two Eating Disorders Specialized Service
Diagnoses of eating disorders	0	0	292
Anorexia nervosa restrictive	0	0	28.8%, *n* = 84
Anorexia Nervosa purgative	0	0	11%, *n* = 32
Bulimia nervosa	0	0	22.9%, *n* = 67
Binge eating disorder	0	0	13%, *n* = 38
OSFED	0	0	24.3%, *n* = 71

*OSFED, other specified feeding or eating disorders*.

As you can see in Table 1, sample 1 was composed of 153 university female students with ages ranging from 18 to 25 years, with a mean of 21.06 years (*SD* = 2.12) who accepted and signed the informed consent. Regarding marital status, 60.7% (*n* = 95) were single, separated, or divorced, and 39.3% (*n* = 58) were living as a couple or married. Sample 2 was composed of 122 participants with obesity. The participants were recruited from the Bariatric Surgery Service of four hospitals in the city of Valencia (Spain). Convenience sampling was used to choose the participants from the waitlist for a bariatric surgery operation. The inclusion criteria were participants who were candidates for obesity surgery and had a BMI above 31 who accepted and signed the informed consent. The exclusion criteria were moderate or severe intellectual disability, diagnosis of an eating disorder, schizophrenia, and bipolar disorders. Most of the sample (64.8%, *n* = 79) is composed of women. The mean age of the sample was 47 years (*SD* = 9.89; range 17–68). The mean body mass index (BMI) was 44.41 (*SD* = 6.03; range 31.59–61.67). Regarding marital status, 60.3% (*n* = 74) were living as a couple or married, and 39.7 (*n* = 48) were single, separated, or divorced. Regarding the level of studies, 43.44% (*n* = 53) had primary studies, 41% (*n* = 50) had secondary studies, 13.11% (*n* = 16) had university studies, and 2.45% (*n* = 3) had no studies. As for medical comorbidity, 77.5% (*n* = 93) of the participants in the study had medical diseases related to morbid obesity, and 9.8% (*n* = 12) of the total sample had a diagnosis of a mental disorder related to anxiety or mood. Sample 3 consisted of 292 participants diagnosed with ED from two Public Mental Health services specialized in ED in Spain. The inclusion criterion was patients who met the DSM-5 criteria for ED. The exclusion criteria were moderate or severe intellectual disability, schizophrenia, and bipolar disorders. Of the 292 participants, 93.5%, *n* = 273, were women, and 6.5% were men, *n* = 19. Regarding diagnoses, 28.8%, *n* = 84, fulfilled the criteria for the AN restrictive diagnosis; 22.9%, *n* = 67, for BN; 11%, *n* = 32, for AN purgative; 13%, *n* = 38, for BED; and 24.3%, *n* = 71, for OSFED. In addition, 22.6%, *n* = 66, had a comorbid diagnosis of personality disorder. The ages of the participants ranged from 12 to 60 years, with a mean age of 24.21 (11.01) years. Regarding their level of studies, 26.4%, *n* = 77, had primary studies; 50.7%, *n* = 148, had secondary studies; and 22.9%, *n* = 67, had higher studies. Regarding marital status, 55.5%, *n* = 162, were single; 41.4%, *n* = 121, were married; and 3.1%, *n* = 9, were separated. All the participants were Caucasian who participated voluntarily and received no compensation.

### Assessments and Measures

#### Sample 1

##### Purpose in Life-10 (PIL-10; García-Alandete et al., 2013)

The PIL is a 10-item Likert-type scale with seven response categories (1–7). It offers a measure of different aspects of meaning in life (for example, “In life I have many definite goals and longings,” “My life is empty and full of despair,” “If I died today, it would seem to me that my life has been very valuable,” “I consider that my ability to find meaning in life is very great,” “I have discovered clear goals and a satisfactory purpose for my life”). We used the Spanish version (García-Alandete et al., 2013), which offers good psychometric properties and high reliability (α = 0.88) and showed excellent reliability in our sample (α = 0.93).

##### Eating Attitudes Test (EAT-40; Garner and Garfinkel, 1979)

The EAT-40 assesses attitudes and behaviors associated with ED. The Spanish version has 40 items organized in three factors and answered on a 6-point Likert scale: (a) Diet and concern about food; (b) Perceived social pressure and discomfort with food; (c) Psychobiological disorders. The instrument offers good psychometric properties and high reliability in patients with AN (α = 0.93) and BN (α = 0.92) in its Spanish version (Castro et al., 1991). In our sample, it showed excellent reliability (α = 0.90).

##### Beck Depression Inventory-II (BDI-II; Beck et al., 1996)

This inventory consists of 21 items with four response alternatives (0–4) that evaluate depressive symptoms. It offers good psychometric properties in its Spanish version (Sanz et al., 2005). In our sample, it presented adequate reliability (α = 0.93).

##### Multidimensional Body-Self Relations Questionnaire-Appearance Scales (MBSRQ-AS 34; Cash, 2000)

The MBSRQ-AS is a self-report composed of five scales with good psychometric properties that assess beliefs and feelings of satisfaction or dissatisfaction with one's appearance. For the present study, we used the Body Areas Satisfaction scale, which consists of nine items and assesses satisfaction or dissatisfaction with specific body areas and attributes (face, hair, lower torso, mid torso, upper torso, muscle tone, weight, height, overall appearance) (Cash, 2000). Each item is scored on a 5-point scale (from 1: “Very dissatisfied” to 5: “Very satisfied”). The Spanish version (Roncero et al., 2015) showed good reliability (α = 0.84). It presents adequate psychometric properties in our sample (α = 0.86).

##### Difficulties in Emotional Regulation Scale (DERS) (Gratz and Roemer, 2004)

This scale assesses emotional regulation difficulties in adults. The Spanish validation of the DERS is made up of 28 elements with a Likert scale (five response levels) (Hervás and Jódar, 2008). For the present study, we chose the emotional dysregulation subscale. The Spanish version of the DERS offered good psychometric properties (α = 0.91) and showed excellent internal consistency in our sample (α = 0.95).

In sample 2, we used several of the assessment instruments described previously: PIL-10, DERS, and MBSRQ-AS. In addition:

##### Structured Clinical Interview for DSM5-Clinical Version (SCID-CV, First et al., 2015)

This is an interview for the main DSM-5 diagnoses (American Psychiatric Association, 2013).

##### Structured Clinical Interview for Personality Disorders DSM-5 (SCID-PD, First et al., 2016)

This is an interview for the diagnosis of personality disorder, based on the DSM-5.

##### Bulimic Test of Edinburgh (BITE, Henderson and Freeman, 1987)

The BITE is a 33-item self-report measure designed to identify subjects with symptoms of BN or BED. The BITE consists of two subscales: the symptom scale, which measures the frequency of symptoms; and the severity scale, which provides an index of the severity of binging and purging behavior. The items on the symptom subscale have a dichotomous format (yes/no), whereas the items on the severity subscale have a Likert-type response format (with 5 or 7 options). The questionnaire offers cutoff points according to levels of severity: (a) a score of 20 or more indicates a highly disordered eating pattern and the presence of binge-eating; (b) a score of 10 to 19 suggests an unusual eating pattern, and a score between 15 and 19 may reflect a subclinical group of binge-eaters, either in the initial stages of the disorder or recovered bulimics; and (c) a score below 10 indicates a non-altered food pattern. In the present study, we obtained adequate reliability (α = 0.79) for the BITE.

##### The Brief Symptoms Inventory (BSI-18; Derogatis, 2001)

The BSI-18 is a self-applied test that consists of 18 items referring to physical, anxious, and depressive symptoms, with responses given on a 4-point Likert scale ranging from 0 (not at all) to 4 (very much). It is made up of three subscales: Depression (six items), Somatization (seven items), and Anxiety (six items). For our study, we only used the depression subscale of the Spanish version of the BIS (Andreu et al., 2008). The depression scale showed adequate reliability (α = 0.79) in the original version, and adequate reliability indices (α = 0.86) were obtained in our data.

In sample 3, we used several of the assessment instruments described previously: SCID-CV and SCID-PD (First et al., 2015, 2016), PIL-10 (García-Alandete et al., 2013), DERS (Gratz and Roemer, 2004), MBSRQ-AS (Cash, 2000), and EAT (Garner and Garfinkel, 1979). In addition:

##### Borderline Symptom List-23 (Bohus et al., 2008)

This is a self-report that assesses the main symptoms of BPD. It is made up of 23 Likert-type items (five response levels). Higher scores on the BSL-23 indicate more severe BPD symptoms. For the present study, we used the Spanish version of the BSL-23 (Soler et al., 2013), which offered good psychometric properties (α = 0.93) and showed excellent internal consistency in our sample (α = 0.95).

##### Beck Hopelessness Scale (Beck et al., 1974)

This is a self-report that assesses negative expectations and attitudes about the future and hopelessness. It is a dichotomous scale containing 20 items (true–false). The scale shows adequate psychometric properties in the Spanish version (Viñas et al., 2004). For our scores, the reliability was adequate (α = 0.89).

### Procedure

In sample 1, participants filled out the questionnaires during their regular school day. Regarding samples 2 and 3, first, an individual evaluation session was carried out to establish the diagnosis using the SCID-CV and SCID-PD. Subsequently, the participants completed the questionnaires. The diagnostic interviews were carried out by a clinical psychologist, with more than 10 years of experience in the evaluation and treatment of ED.

### General Statistical Procedure

The statistical procedure was similar for all three samples. First, descriptive statistics and zero-order correlations (Pearson's coefficient) were calculated for the variables. Second, a multiple mediation analysis was performed. To test the mediational models, we calculated three effects (Frazier et al., 2004): (a) the direct effect, where the outcome variable (EAT, BAS, BDI in sample 1; BITE, BAS, BIS in sample 2; and EAT, BAS, HS, BSL in sample 3) is regressed on the predictor (DERS); (b) the indirect effect, which consists of two paths: first, the mediator (PIL) is regressed on the predictor variable (DERS), and second, the outcome variable (EAT, BAS, BDI in sample 1; BITE, BAS, BIS in sample 2; and EAT, BAS, HS, BSL in sample 3) is regressed on both the predictor (DERS) and the mediator (PIL); (c) third, the total effect, which is the sum of the direct and indirect effect. If the indirect effect is significantly smaller than the direct effect, the data suggest partial mediation. In addition, we tested the significance of the mediated effect with the CIs using bias-corrected bootstrap because the sampling distribution of the indirect effect is asymmetric. We used the Delta method SEs and bias-corrected percentile bootstrap (10,000 replications), and we calculated the variance explained (adjusted R^2^) by the mediation. The calculations and the mediation model were performed with the statistical program JASP Team (2020).

## Results

**Figure 1:**
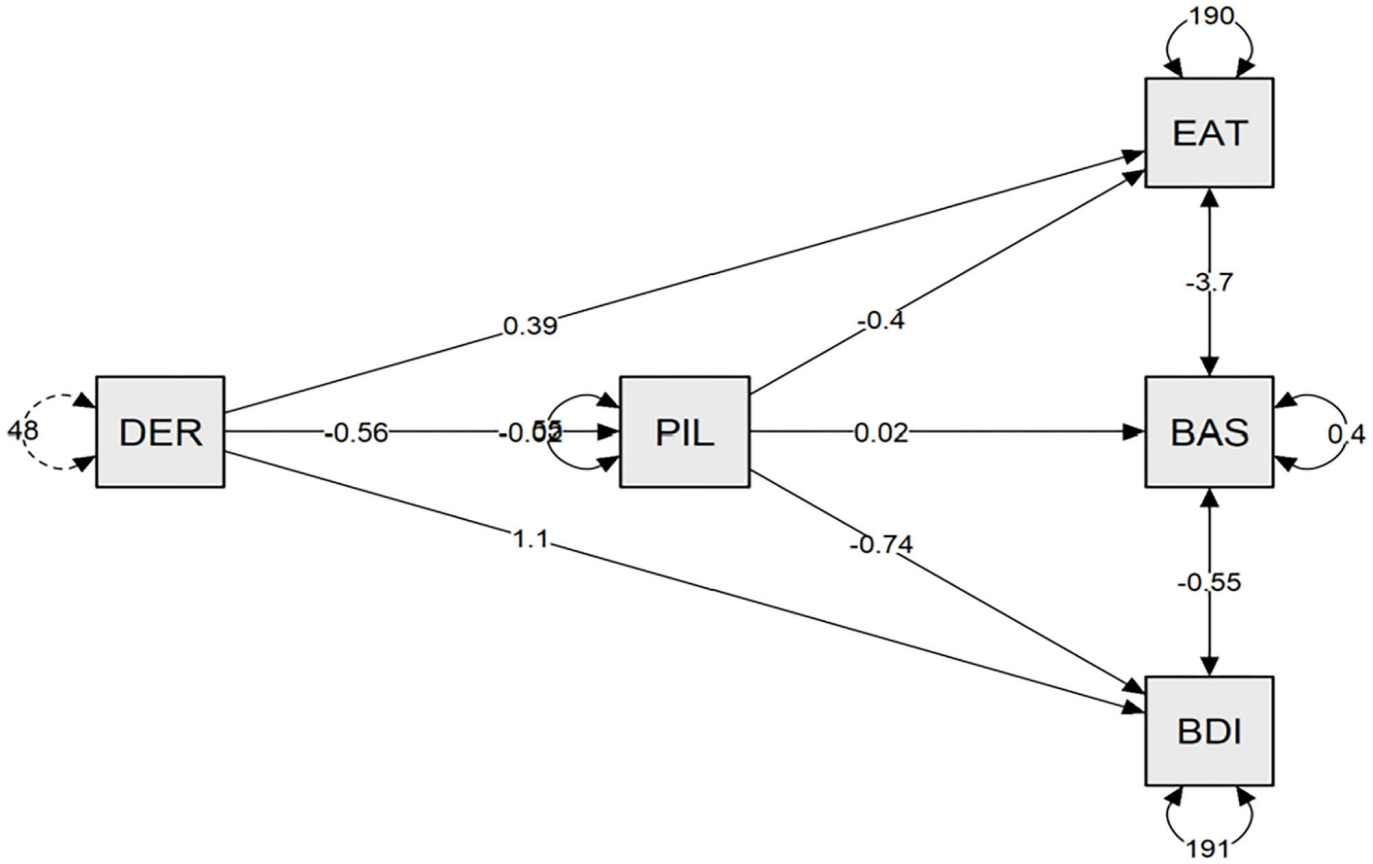
The meaning in life is a mediating variable between the emotional deregulation and binge eating disorder psychopathology, and depression in young adult women without eating disorders. DER, emotional deregulation; PIL, purpose in life; BDI, Beck Depression Inventory; BAS, body area satisfaction.

As you can see in Table 2, in the sample composed by participants without ED, meaning in life had a high and negative correlation with depression symptoms, a moderate and negative correlation with emotional dysregulation, and a moderate and positive correlation with body satisfaction. Furthermore, meaning in life had a low and negative correlation with negative attitudes and behaviors toward the body. As Table 3 reveals, meaning in life showed a multiple mediation effect between emotional dysregulation and the psychopathology of eating disorders (direct effect β = 0.390, *p* < 0.05) (indirect effect β = 0.227, *p* < 0.05) (R^2^ = 0.13), body satisfaction (direct effect β = −0.017, *p* < 0.05) (indirect effect β = −0.013, *p* < 0.01) (R^2^ = 0.16), and depression symptoms (direct effect β = 1.112, *p* < 0.001) (indirect effect β = 0.414, *p* < 0.001) (R^2^ = 0.42). As Figure 1 shows, the relationship between meaning in life and emotional dysregulation was negative, and the relationships between meaning in life and eating disorder symptoms and depression were negative. Finally, the relationship between meaning in life and body satisfaction was positive.

**Table 2:** Mean and zero-order correlations for the variables in young women.

	**M (SD)**	**2**	**3**	**4**	**5**
1. Meaning in life (PIL)	56.63 (8.27)	−0.46^**^	−0.29^**^	0.37^**^	−0.53^**^
2. Emotional dysregulation	16 (7.01)		0.27^**^	−0.34^**^	0.57^**^
3. Eating attitude test	11.59 (15.08)			−0.50^**^	0.33^**^
4. Body areas satisfaction	3.32 (0.67)				−0.28^**^
5. Depression Inventory	12.88 (.97)				

*PIL, Purpose in life*.

** *p < 0.01*.

**Table 3:** Multiple mediation model of meaning in life between emotional dysregulation and the psychopathology of eating disorders in young adult women without eating disorders.

	**Estimate**	**SE**	***z*-Value**	***p***	**95% CI**	
					**Lower**	**Upper**
**DER-PIL-EAT**						
Total effect	0.617	0.178	3.472	<0.001	0.269	0.965
Direct effect	0.390	0.191	2.042	0.041	0.016	0.764
Indirect effect	0.227	0.097	2.347	0.019	0.037	0.417
**DER-PIL-BAS**						
Total effect	−0.031	0.008	−3.963	<0.001	−0.046	−0.016
Direct effect	−0.017	0.008	−2.066	0.039	−0.034	−8.949e−4
Indirect effect	−0.013	0.004	−2.970	0.003	−0.022	−0.005
**DER-PIL-BDI**						
Total effect	1.527	0.189	8.090	<0.001	1.157	1.896
Direct effect	1.112	0.205	5.439	<0.001	0.712	1.513
Indirect effect	0.414	0.111	3.728	<0.001	0.196	0.632

*DER, emotional deregulation; PIL, purpose in life; EAT, eating attitude test; BAS, body areas satisfaction; BDI, Beck Depression Inventory*.

**Figure 2:**
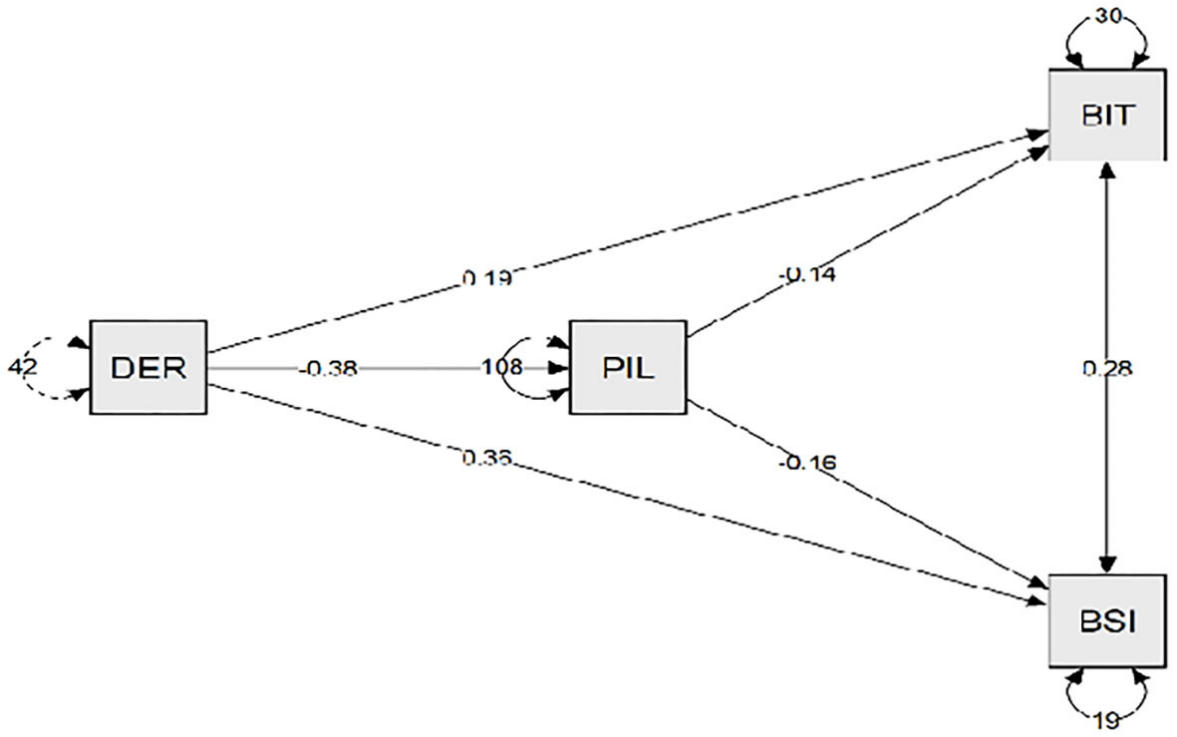
The meaning in life is a mediating variable between the emotional deregulation and binge eating disorder psychopathology, and depression in participants with overweight. DER, emotional deregulation; PIL, purpose in life; BIT, Bulimic Inventory Test Edinburgh; BSI, depression subscale of the Brief Symptoms Inventory.

Regarding the sample composed by participants with obesity, Table 4 shows that meaning in life had a moderate and negative correlation with BN and BED symptoms and with depression symptoms. Furthermore, meaning in life had a low and negative correlation with emotional dysregulation. On the other hand, meaning in life had a moderate and positive correlation with body satisfaction. Emotional dysregulation was not associated with body satisfaction, and so it was excluded from the mediation model. As Table 5 reveals, meaning in life showed a multiple mediation effect between emotional dysregulation and the psychopathology of BN and BED symptoms (direct effect β = 0.194, *p* < 0.01) (indirect effect β = 0.054, *p* < 0.05) (R^2^ = 0.15) and depression symptoms (direct effect β = 0.357, *p* < 0.001) (indirect effect β = 0.063, *p* < 0.05) (R^2^ = 0.35). As Figure 2 shows, the relationship between meaning in life and emotional dysregulation was negative, and the relationships between meaning in life and BN and BED symptoms and depression were negative.

**Table 4:** Mean and zero-order correlations for the variables in participants with obesity.

	**M (SD)**	**2**	**3**	**4**	**5**
1. Meaning in life (PIL)	53.88 (10.74)	−0.23^*^	−0.30^**^	0.41^**^	−0.42^**^
2. Emotional dysregulation	14.48 (6.54)		0.27^**^	−0.15	0.55^**^
3. BITE	10.27 (5.88)			−0.40^**^	0.22^*^
4. Body Satisfaction Scale	2.56 (0.57)				−0.28^**^
5. Depression (BSI)	4.42 (5.44)				–

*PIL, purpose in life; BITE, Bulimic Inventory Test Edinburgh; BSI, Brief Symptoms Inventory*.

* *p < 0.05*,

** *p < 0.01*.

**Table 5:** Multiple mediation model of meaning in life between emotional dysregulation and the bulimic and binge eating disorders symptoms and depression in participants with obesity.

	**Estimate**	**SE**	***z*-Value**	***p***	**95% CI**	
					**Lower**	**Upper**
**DER-PIL-BITE**						
Total effect	0.247	0.078	3.157	<0.001	0.039	0.413
Direct effect	0.194	0.08	2.485	<0.01	0.025	0.362
Indirect effect	0.054	0.027	1.960	<0.05	0.006	0.154
**DER-PIL-Depression (BSI)**						
Total effect	0.420	0.065	6.453	<0.001	0.238	0.572
Direct effect	0.357	0.062	5.719	<0.001	0.159	0.542
Indirect effect	0.063	0.028	2.247	<0.05	0.010	0.165

*PIL, purpose in life; BITE, Bulimic Inventory Test Edinburgh; BSI, Brief Symptoms Inventory*.

**Figure 3:**
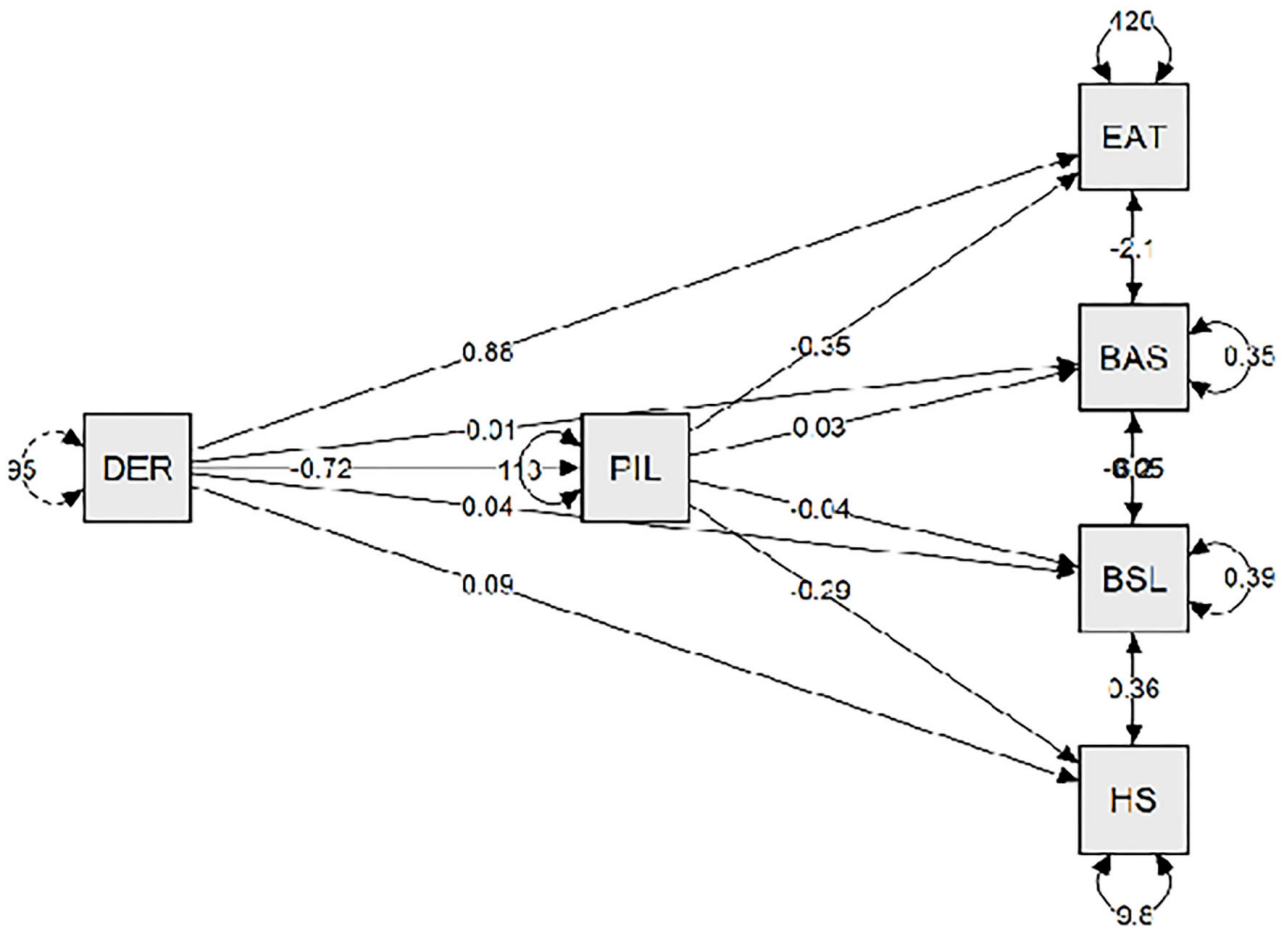
The meaning in life is a mediating variable between the emotional deregulation and the main symptoms of eating disorders. DER, emotional deregulation; PIL, purpose in life; EAT, eating attitude test; BAS, body area satisfaction; BSL, borderline symptom list; HS, hopelessness scale.

In the sample composed by participants with ED diagnoses (see Table 6), meaning in life had a high and negative correlation with emotional dysregulation, hopelessness, and BPD symptoms and a high and positive association with body satisfaction. Moreover, meaning in life had a moderate and negative correlation with negative attitudes and behaviors toward the body and food. As Table 7 reveals, meaning in life showed a multiple mediation effect between emotional dysregulation and negative attitudes toward food (direct effect β = 0.884, *p* < 0.001) (indirect effect β = 0.252, *p* < 0.007) (R^2^ = 0.24), body satisfaction (direct effect β = −0.033, *p* < 0.05) (indirect effect β = −0.021, *p* < 0.001) (R^2^ = 0.37), borderline symptoms (direct effect β = 0.040, *p* < 0.001) (indirect effect β = 0.025, *p* < 0.001) (R^2^ = 0.58), and hopelessness (direct effect β = 0.211, *p* < 0.001) (indirect effect β = 0.087, *p* < 0.001) (R^2^ = 0.65). As Figure 3 shows, the relationship between meaning in life and emotional dysregulation was negative, and the relationships between meaning in life and negative attitudes toward food, hopelessness, and borderline symptoms were negative. However, the relationship between meaning in life and body satisfaction was positive.

**Table 6:** Mean and zero-order correlations for the variables in participants with ED.

	**M (SD)**	**2**	**3**	**4**	**5**	**6**
1. Meaning in life (PIL)	43.88 (12.51)	−0.50^**^	−0.36^**^	0.51^**^	−0.60^**^	−0.70^**^
2. Emotional dysregulation	24.29 (9.84)		0.44^**^	−0.45^**^	0.68^**^	0.55^**^
3. Eating attitude test	38.96 (23.11)			−0.40^**^	0.52^**^	0.40^**^
4. Body areas satisfaction	2.62 (0.74)				−0.52	−0.55^**^
5. Borderline symptoms	1.17 (0.97)					0.66^**^
6. Hopelessness Scale	6.26 (5.30)					–

*PIL, purpose in life; BSL, borderline symptoms list; HS, Hopelessness Scale*.

** *p < 0.01*.

**Table 7:** Multiple mediation model of meaning in life between emotional dysregulation and the psychopathology of eating disorders in participants with eating disorders.

	**Estimate**	**SE**	***z*-Value**	***p***	**95% CI**	
					**Lower**	**Upper**
**DER-PIL-EAT**						
Total effect	1.136	0.138	8.220	<0.001	0.846	1.404
Direct effect	0.884	0.163	5.437	<0.001	0.555	1.225
Indirect effect	0.252	0.093	2.718	0.007	0.087	0.437
**DER-PIL-BAS**						
Total effect	−0.054	0.004	−7.563	<0.001	−0.043	−0.024
Direct effect	−0.033	0.005	−2.688	0.007	−0.022	−0.003
Indirect effect	−0.021	0.003	−6.336	<0.001	−0.027	−0.016
**DER-PIL-BSL**						
Total effect	0.065	0.005	13.607	<0.001	0.055	0.075
Direct effect	0.040	0.005	8.162	<0.001	0.031	0.050
Indirect effect	0.025	0.004	6.874	<0.001	0.019	0.033
**DER-PIL-HS**						
Total effect	0.298	0.029	10.158	<0.001	0.232	0.355
Direct effect	0.211	0.025	8.494	<0.001	0.165	0.256
Indirect effect	0.087	0.025	3.515	<0.001	0.039	0.142

*DER, emotional deregulation; PIL, purpose in life; EAT, eating attitude test; BAS, body areas satisfaction; BSL, borderline symptom list; HS, Hopelessness Scale*.

## Discussion

The aim of our study was to test the mediating role of meaning in life in the relationship between emotional dysregulation and the psychopathology of ED in three different samples of participants: young women participants, participants with morbid obesity, and participants with a diagnosis of ED.

In the first sample, meaning in life showed a partial mediation effect between emotional dysregulation and the ED psychopathology, body satisfaction, and depression. We would like to highlight that meaning in life showed a negative association with the ED psychopathology, a positive association with body satisfaction, and a negative association with depression, and these associations were similar in size to the associations with emotional dysregulation, an important variable in the etiology of ED. These results are similar to previous studies with samples without ED, where they found that meaning in life was negatively associated with ED symptoms in adolescents (Gongora, 2014) and positively associated with healthy eating (Brassai et al., 2015). However, in our study we have taken a step further by showing that meaning in life is a mediator variable in the relationship between the risk factor of emotional dysregulation and other risk factors for ED, such as negative attitudes toward food, body satisfaction, and depression in young women. This result is important because negative affect has been found to be a predictor of the onset of all types of EDs, including AN, BN, BED, and purging disorder, because it can decrease appetite, leading to unhealthy weight loss and increasing the likelihood of unhealthy weight control behaviors (Stice et al., 2017).

Regarding the second sample, participants with obesity, the results suggest that meaning in life was a mediating variable between emotional dysregulation and BN and BED symptoms and depression. As in the young women participants, meaning in life was moderately and positively associated with body satisfaction. We selected participants with obesity because it is a risk factor for developing ED and it has high comorbidity with BN and BED (Dixon et al., 2003). When we designed the study, we thought that if meaning in life is a mediating variable in the ED psychopathology, its mediating role should be confirmed with a sample with high vulnerability to ED, such as people with obesity. This hypothesis has been confirmed in the present study. Moreover, we want to emphasize that meaning in life was highly and negatively associated with depression. This result is important because participants with morbid obesity have high comorbidity with depression (Dixon et al., 2003).

Regarding the third sample, participants diagnosed with ED, meaning in life was a significant mediator between emotional dysregulation and the main symptoms of ED, such as a negative attitude toward food and the body, body satisfaction, hopelessness, and borderline symptoms. We want to highlight that meaning in life has a greater association with body satisfaction than with emotional dysregulation and negative attitudes and behaviors toward food and the body. Furthermore, these results agree with previous studies indicating that meaning in life is an important variable in participants with ED (Marco et al., 2020b), as well as other qualitative studies on the analysis of recovery criteria in ED that found meaning in life and purpose to be important components of ED recovery (de Vos et al., 2017).

In all three samples, meaning in life was highly and negatively associated with depression and positively associated with body satisfaction, and several studies have found that body dissatisfaction and depressive symptoms are the main predictors of ED (e.g., Stice et al., 2011). In this regard, Troop (2016) suggests that negative affect about a loss is a precipitating factor of the ED, and Gulliksen et al. (2017) suggest that the initiation of the ED is an attempt to control the negative emotions, family environment, and challenges patients have experienced at any given moment in their lives. Thus, from the MMMED perspective, we can suggest that, in some patients, the ED symptoms could be a dysfunctional strategy to achieve a new meaning in life once the previous meaning in life has been threatened or lost at a certain time in their lives. If the new meaning in life is focused on the ED values, aims, and beliefs, this keeps patients from discovering an authentic, genuine, and individual meaning in life. If the person with ED does not discover an authentic meaning in life, it could lead to a state of hopelessness, a lack of identity, and low quality of life. In this regard, studies have found that people with ED have lower levels of meaning in life than recovered patients (de la Rie et al., 2007), problems with their identity (Stein and Corte, 2007), feelings of hopelessness (Robinson et al., 2015), and poor quality of life (Tomba et al., 2017). In a recent study, Wetzler et al. (2020) confirmed that connectedness, hope, and optimism about the future, identity, meaning in life, and empowerment are important components of recovery in ED participants. Thus, patients indicated that they found meaning in their lives from ED, and finding actual meaning in life and important goals and values beyond ED is a very important part of recovery.

Although the absence of meaning in life can be a symptom included in several mental disorders (e.g., major depressive disorder, adjustment disorders, BPD, etc.), meaning in life is a different construct from depression for several reasons. Meaning in life is a transdiagnostic construct composed of the sense of coherence, purpose, and importance of our life that is not necessarily psychotogenic. Moreover, regarding patients with depressive symptoms, Frankl (2006) differentiated between noogenic depression (noos = meaning) and endogenous depression, to highlight the patients whose depression was caused by the absence of meaning in life. Frankl states that noogenic depression is characterized by an existential vacuum, absence of meaning in life, boredom, frustration, distress, anxiety, and aggressiveness, and that its etiology would be different from that of endogenous depressions (e.g., neurobiological disturbances, early depressive schemes), indicating that around 20% of depressions are noogenous. Regarding participants with adjustment disorder, several studies have found that meaning-making was an independent and buffering factor in adjustment in participants with depression and anxiety disorders (e.g., Marco et al., 2020a).

Our results could have several clinical implications. One of the main difficulties in treating ED is the rejection and resistance to change these patients usually show, often because they may not recognize ED symptoms as a problem, thus making collaboration in treatment difficult (Macdonald et al., 2012). In a qualitative study, Nordbø et al. (2012) asked AN patients specifically about what makes them not want to recover, and they found that having AN can evoke positive feelings, such as feelings of security or the feeling that there is meaning and purpose in life. These feelings compensated for the negative consequences of AN, and so the desire to recover decreased. Thus, taking into account the studies that suggest that meaning and purpose in life are important factors in the recovery process, it is possible that if we orient psychotherapy toward values and goals related to authentic and genuine sources of meaning, we can increase the motivation toward recovery. Second, our results could suggest the need to add meaning-centered therapy for ED participants with low meaning in life. Qualitative research carried out from the patients' perspective indicated that they need to be treated as a “whole person” (Rance et al., 2017), preferring treatments that take their psychological and social needs into account, and they stated that rigid treatments focused on weight and food did not work for them (Westwood and Kendal, 2012).

We want to emphasize that the MMMED does not suggest that all patients with ED have low meaning in life or that it is necessary to intervene in the meaning in life in all patients. On the contrary, in most cases the ED will develop and be maintained by other factors, such as self-esteem, depression, body dissatisfaction, and perfectionism (Fairburn, 2008). The MMMED suggests that only a percentage of participants will have problems with meaning in life. However, in cases where the absence of meaning in life is a problem, we suggest adapting the current psychotherapies, mainly cognitive behavioral therapy or dialectical behavioral therapy (Linehan, 2015), by adding a treatment component focused on meaning-centered therapy. Thus, meaning-centered therapy for ED with low meaning in life could focus on the following aims: (1) awareness of their absence of meaning in life because their life is oriented toward dysfunctional goals and values (e.g., weight control, food control, perfectionism, etc.); (2) psychoeducation about meaning in life as a protective factor against ED symptoms; (3) learning to recognize the potential sources of meaning, as well as discovering situations from the past that involved moments of fulfillment; (4) learning to discover authentic vital purpose and goals; (5) modifying their current aims and goals to follow their authentic values. Meaning-centered therapy could be added to the therapies that are efficacious in improving emotional regulation problems in ED. Meta-analytic studies found that meaning-centered therapy was primarily effective in improving general quality of life and meaning in life; reducing psychological stress and negative affect; and improving social relationships, self-efficacy, hope, hopelessness, and optimism (Vos and Vitali, 2018). To date, no studies have analyzed whether a treatment component focused on meaning in life added to CBT would be effective in improving emotional dysregulation, depression, and body dissatisfaction in people with ED. Thus, future research should analyze this.

Regarding the strategies for ED prevention, meta-analysis studies found that media literacy was effective for reducing ED risk factors up to 30 months after the intervention in both females and males, and that multicomponent and self-esteem enhancement interventions were effective only in females. Moreover, the cognitive dissonance intervention was superior to controls in reducing ED behaviors up to 3 years post-intervention. In the same way, healthy weight interventions and CBT interventions improved ED risk factors (Long et al., 2017). However, it is important to emphasize that the incidence of ED has not decreased in the past 50 years (Hoek, 2016), and so, in addition to the previously mentioned prevention strategies, an intervention could be carried out on other variables that have been shown to buffer the risk factors for ED. Our results suggest that interventions aimed at discovering authentic and genuine meaning in life could be a strategy for the prevention of risk factors: depression, body dissatisfaction, emotional dysregulation, and negative attitudes toward food and the body. In this regard, there are programs aimed at adolescents and young adults that have been shown to improve meaning in life. For example, Luz et al. (2017) found that, after the intervention to increase meaning in life in adolescents, the perception of meaning in life increased and negative affect decreased, providing evidence supporting the effectiveness of the intervention. Cheng et al. (2015) carried out a program with university students in China where therapy focused on meaning in life was found to increase psychological well-being. Thus, future research needs to analyze whether a meaning-focused intervention can be effective in reducing risk factors for ED in the general population.

The present study has some limitations: first, all the studies are cross-sectional, which means that we cannot speak of causality between variables. For this reason, the results obtained should be considered in terms of correlates rather than causal risk factors. More research is needed to replicate the present study using a longitudinal design. Another limitation is that, although meaning in life was a mediator variable of ED psychopathology, the size of the mediation was high for hopelessness and borderline symptoms, moderate for depression symptoms (range between 0.35 and 0.45), low to moderate for body satisfaction (range between 0.16 and 0.37), and low for eating disorder symptoms (range between 0.13 and 0.24). These results indicate that other variables that we have not included in the present study may influence the relationships between emotional dysregulation, meaning in life, and ED psychopathology, such as perfectionism. All the studies were carried out with Spanish participants, and so these results are only generalizable to countries similar to the Spanish culture. Another limitation of our study is that we did not assess the exogenous or endogenous origins of obesity in the participants who suffered from obesity. We understood that obesity could be a consequence of ED, and that meaning in life is a mediator of ED psychopathology. Thus, future research should analyze this distinction to determine whether meaning in life buffers the association between ED psychopathology and obesity symptoms. Finally, another limitation of this research is that we cannot compare the three samples because the dependent variables were measured with different scales. For example, to assess depression, we used the BDI in the non-clinical sample, the BIS questionnaire in the participants with obesity, and the HS in the sample with ED. In the same way, to assess ED symptoms, we used the EAT in the non-clinical sample and the sample with ED, but in the sample with obesity, we used the BITE. Thus, future research will have to carry out a new study with three samples with different ED psychopathology severity, but assessed with the same measures, to compare the results of the three samples by performing a multi-group model (e.g., SEM) that includes all the participants and assessing the role of the diagnostic subtype with invariance tests.

In conclusion, our study suggests that meaning in life is a mediating variable between emotional dysregulation and the main risk factors for ED in participants with ED, participants with obesity, and young female participants. These results suggest the importance of considering meaning in life as a relevant variable in the onset and maintenance of people with ED.

## Data Availability Statement

The raw data supporting the conclusions of this article will be made available by the authors, without undue reservation.

## Ethics Statement

The studies involving human participants were reviewed and approved by the Catholic University of Valencia's ethics committee prior to its implementation (research code UCV2013–2014/0023). Written informed consent to participate in this study was provided by the participants' legal guardian/next of kin.

## Author Contributions

JM did the experimental design, performed the analyses, and wrote the manuscript. MC and CM recruited the sample and wrote the manuscript. RB, VG, and SP designed the research and reviewed the final manuscript. All authors contributed to the article and approved the submitted version.

## Funding

Funding for the study was provided by (R + D + I) Projects of the State Programs Oriented to the Challenges of Society, within the framework of the State Research Plan Scientific and Technical and Innovation, with Code: PID2019-111036RB-I00, from Ministry of Science and Innovation of Spain.

## Conflict of Interest

The authors declare that the research was conducted in the absence of any commercial or financial relationships that could be construed as a potential conflict of interest.

## References

[B1] Aldao A. Nolen-Hoeksema S. Schweizer S. (2010). Emotion-regulation strategies across psychopathology: a meta-analytic review. Clin. Psychol. Rev. 30, 217–237. 10.1016/j.cpr.2009.11.004 20015584

[B2] Allen K. Byrne S. Oddy W. Crosby R. (2013). DSM-IV-TR and DSM-5 eating disorders in adolescents; Prevalence, stability, and psychosocial correlates in a population-based sample of male and female adolescents. J. Abnorm. Psychol. 122, 720–732. 10.1037/a0034004 24016012

[B3] American Psychiatric Association (2013). Diagnostic and Statistical Manual of Mental Disorders, 5th Edn. Washington, DC: APA.

[B4] Andreu Y. Galdón M. J. Dura E. Ferrando M. Murgui S. García A.. (2008). Psychometric properties of the Brief Symptoms Inventory-18 (BSI-18) in a Spanish sample of outpatients with psychiatric disorders. Psicothema 20, 844–850. 18940093

[B5] Beck A. T. Steer R. A. Brown G. K. (1996). Manual for the Beck Depression Inventory-II, 2nd Edn. San Antonio, TX: Psychological Corporation.

[B6] Beck A. T. Weissman A. Lester D. Trexler L. (1974). The measurement of pessimism: the hopelessness scale. J. Consult. Clin. Psychol. 42, 861–865. 10.1037/h0037562 4436473

[B7] Black D. W. Goldstein R. B. Mason E. E. (1992). Prevalence of mental disorders in 88 morbidly obese bariatric clinic patients. Am. J. Psychiatry 149, 227–234. 10.1176/ajp.149.2.227 1734744

[B8] Bohus M. Kleindienst N. Limberger M. F. Stieglitz R. D. Domsalla M. Chapman A. L.. (2008). The short version of the Borderline Symptom List (BSL-23): development and initial data on psychometric properties. Psychopathol 42, 32–39. 10.1159/000173701 19023232

[B9] Bowlby C. G. Anderson T. L. Hall M. E. L. Willingham M. M. (2015). Recovered professionals exploring eating disorder recovery: a qualitative investigation of meaning. Clin. Soc. Work J. 43, 1–10. 10.1007/s10615-012-0423-0

[B10] Brassai L. Piko B. F. Steger M. F. (2015). A reason to stay healthy: the role of meaning in life in relation to physical activity and healthy eating among adolescents. J. Health Psychol. 20, 473–482. 10.1177/1359105315576604 25903235

[B11] Brone R. J. Fisher C. B. (1988). Determinants of adolescent obesity: a comparison with anorexia nervosa. Adolescence 23, 155–169. 3289309

[B12] Cash T. F. (2000). The MBSRQ Users' Manual, 3rd Edn. Available online at: www.body-images.com.Cash

[B13] Castro J. Toro J. Salamero M. Guimerá E. (1991). The eating attitudes test: validation of the Spanish version. Psychol. Assess. 7, 175–190.

[B14] Cheng M. Hasche L. Huang H. Su X. S. (2015). The effectiveness of a meaning-centered psychoeducational group intervention for Chinese college students. J. Soc. Behav. Pers. 43, 741–756. 10.2224/sbp.2015.43.5.741

[B15] de la Rie S. Noordenbos G. Donker M. van Furth E. (2007). The patient's view on quality of life and eating disorders. Int. J. Eat. Disord. 40, 13–20. 10.1002/eat.20338 16941625

[B16] de Vos J. A. LaMarre A. Radstaak M. Bijkerk C. A. Bohlmeijer E. T. Westerhof G. J. (2017). Identifying fundamental criteria for eating disorder recovery: a systematic review and qualitative meta-analysis. J. Eat. Disord. 5:34. 10.1186/s40337-017-0164-0 29118983 PMC5664841

[B17] Derogatis L. R. (2001). Brief Symptom Inventory (BSI)-18. Administration, Scoring and Procedures Manual. Minneapolis, MN: NCS Pearson, Inc.

[B18] Dixon J. B. Dixon M. E. O'Brien P. E. (2003). Depression in association with severe obesity: changes with weight loss. Arch. Intern. Med. 163, 2058–2065. 10.1001/archinte.163.17.2058 14504119

[B19] Fairburn C. G. (2008). Cognitive Behavior Therapy and Eating Disorders. New York, NY: Guilford Press.

[B20] First M. B. Williams J. B. Benjamin L. S. Spitzer R. L. (2016). Structured Clinical Interview for DSM-5^®^ Personality Disorders. Washington, DC: American Psychiatric Association Publishing.

[B21] First M. B. Williams J. B. W. Karg R. S. Spitzer R. L. (2015). Structured Clinical Interview for DSM-5 Disorders—Clinician Version (SCID-5-CV). Arlington, VA: American Psychiatric Association.

[B22] Fox A. P. Leung N. (2009). Existential well-being in younger and older people with anorexia nervosa—a preliminary investigation. Eur. Eat. Disord. Rev. 17, 24–30. 10.1002/erv.895 18759376

[B23] Frankl V. E. (2006). The Unheard Cry for Meaning. Psychotherapy and Humanism. Boston, MA: Beacon Press.

[B24] Frazier P. Tix A. P. Barron K. E. (2004). Testing moderator and mediator effects in counseling psychology research. J. Couns. Psychol. 51, 115–134. 10.1037/0022-0167.51.1.115

[B25] García-Alandete J. Rosa E. Sellés P. (2013). Estructura factorial y consistencia interna de una version española del Purpose-In-Life Test [Factorial structure and internal consistency of a Spanish version of the Purpose-In-Life Test]. Univ. Psychol. 12, 517–530. 10.11144/Javeriana.UPSY12-2.efci

[B26] Garner D. M. Garfinkel P. E. (1979). The eating attitudes test: an index of the symptoms of anorexia nervosa. Psychol. Med. 9, 273–279. 10.1017/S0033291700030762 472072

[B27] Garrett C. J. (1997). Recovery from anorexia nervosa: a sociological perspective. Int. J. Eat. Disord. 21, 261–272. 10.1002/(SICI)1098-108X(199704)21:3<261::AID-EAT6>3.0.CO;2-I 9097199

[B28] Gongora V. (2014). Satisfaction with life, well-being, and meaning in life as protective factors of eating disorder symptoms and body dissatisfaction in adolescents. Eat. Disord. 22, 435–449. 10.1080/10640266.2014.931765 24983397

[B29] Gratz K. L. Roemer L. (2004). Multidimensional assessment of emotion regulation and dysregulation: development, factor structure, and initial validation of the difficulties in emotion regulation scale. J. Psychopathol. Behav. Assess. 26, 41–54. 10.1023/B:JOBA.0000007455.08539.94

[B30] Gross J. J. (1998). The emerging field of emotion regulation: an integrative review. Rev. Gen. Psychol. 2, 271–299. 10.1037/1089-2680.2.3.271

[B31] Guisado J. A. Vaz F. J. López-Ibor J. J. Inés López-Ibor M. del Río J. Rubio M. A. (2002). Gastric surgery and restraint from food as triggering factors of eating disorders in morbid obesity. Int. J. Eat. Disord. 31, 97–100. 10.1002/eat.1114 11835303

[B32] Gulliksen K. S. Nordbø R. H. Espeset E. M. Skårderud F. Holte A. (2017). Four pathways to anorexia nervosa: patients' perspective on the emergence of AN. Clin. Psychol. Psychother. 24, 846–858. 10.1002/cpp.2050 27726246

[B33] Henderson M. Freeman C. P. L. (1987). A self-rating scale for Bulimia. The BITE. Br. J. Psychiatry 150, 18–24. 10.1192/bjp.150.1.18 3651670

[B34] Hervás G. Jódar R. (2008). The Spanish version of the difficulties in emotion regulation scale. Clín. Salud 19, 139–156.

[B35] Hoek H. W. (2016). Review of the worldwide epidemiology of eating disorders. Curr. Opin. Psychiatry 29, 336–339. 10.1097/YCO.0000000000000282 27608181

[B36] Jarman F. C. Rickards W. S. Hudson I. L. (1991). Late adolescent outcome of early unset anorexia nervosa. Pediatr. Child Health 27, 221–227. 10.1111/j.1440-1754.1991.tb00396.x 1958421

[B37] JASP Team (2020). JASP (Version 0.14.1) [Computer Software]. Available online at: https://jasp-stats.org/

[B38] Linehan M. M. (2015). DBT Skills Training Manual, 3nd Edn. New York, NY: Guilford Press.

[B39] Long K. D. L. Barendregt J. J. Hay P. Mihalopoulos C. (2017). Prevention of eating disorders: a systematic review and meta-analysis. Clin. Psychol. Rev. 53, 46–58. 10.1016/j.cpr.2017.02.001 28214633

[B40] Luz J. M. O. D. Murta S. G. Aquino T. A. A. D. (2017). Intervention for promoting meaning in life in adolescents: evaluation of the process and results. Trends Psychol. 25, 1795–1811. 10.9788/TP2017.4-14En

[B41] Macdonald P. Hibbs R. Corfield F. Treasure J. (2012). The use of motivational interviewing in eating disorders: a systematic review. Psychiatry Res. 200, 1–11. 10.1016/j.psychres.2012.05.013 22717144

[B42] Mallorquí-Bagué N. Vintró-Alcaraz C. Sánchez I. Riesco N. Agüera Z. Granero R.. (2018). Emotion regulation as a transdiagnostic feature among eating disorders: cross-sectional and longitudinal approach. Eur. Eat. Disord. Rev. 26, 53–61. 10.1002/erv.2570 29168283

[B43] Marco J. H. Alonso S. Baños R. (2020a). Meaning-making as a mediator of anxiety and depression reduction during cognitive behavioral therapy intervention in participants with adjustment disorders. Clin. Psychol. Psychother. 10.1002/cpp.2506. [Epub ahead of print]. 32881109

[B44] Marco J. H. Cañabate M. Llorca G. Pérez S. (2020b). Meaning in life moderates hopelessness, suicide ideation, and borderline psychopathology in participants with eating disorders: a longitudinal study. Clin. Psychol. Psychother. 27, 146–158. 10.1002/cpp.2414 31765024

[B45] Marco J. H. Cañabate M. Pérez S. (2019). Meaning in life is associated with the psychopathology of eating disorders: differences depending on the diagnosis. Eat. Disord. 27, 550–564. 10.1080/10640266.2018.1560852 30663525

[B46] Marco J. H. Cañabate M. Pérez S. Llorca G. (2017). Associations among meaning in life, body image, psychopathology, and suicide ideation in Spanish participants with eating disorders. J. Clin. Psychol. 73, 1768–1781. 10.1002/jclp.22481 28419452

[B47] Martela F. Steger M. F. (2016). The three meanings of meaning in life: distinguishing coherence, purpose, and significance. J. Posit. Psychol. 11, 531–545. 10.1080/17439760.2015.1137623

[B48] Micanti F. Iasevoli F. Cucciniello C. Costabile R. Loiarro G. Pecoraro G.. (2017). The relationship between emotional regulation and eating behaviour: a multidimensional analysis of obesity psychopathology. Eat. Weight Disord. 22, 105–115. 10.1007/s40519-016-0275-7 27068173 PMC5334401

[B49] Monell E. Högdahl L. Mantilla E. F. Birgegård A. (2015). Emotion dysregulation, self-image and eating disorder symptoms in University Women. J. Eat. Disord. 3, 1–11. 10.1186/s40337-015-0083-x 26629343 PMC4666164

[B50] Nordbø R. H. Espeset E. M. Gulliksen K. S. Skårderud F. Geller J. Holte A. (2012). Reluctance to recover in anorexia nervosa. Eur. Eat. Disord. Rev. 20, 60–67. 10.1002/erv.1097 21305676

[B51] Pisetsky E. M. Haynos A. F. Lavender J. M. Crow S. J. Peterson C. B. (2017). Associations between emotion regulation difficulties, eating disorder symptoms, non-suicidal self-injury, and suicide attempts in a heterogeneous eating disorder sample. Compr. Psychiatry 73, 143–150. 10.1016/j.comppsych.2016.11.012 27978502 PMC5263187

[B52] Racine S. E. Wildes J. E. (2015). Dynamic longitudinal relations between emotion regulation difficulties and anorexia nervosa symptoms over the year following intensive treatment. J. Consult. Clin. Psychol. 83, 785–795. 10.1037/ccp0000011 25181027 PMC4345157

[B53] Rance N. Moller N. P. Clarke V. (2017). ‘Eating disorders are not about food, they're about life': client perspectives on anorexia nervosa treatment. J. Health Serv. Psychol. 22, 582–594. 10.1177/1359105315609088 26446375

[B54] Robinson P. H. Kukucska R. Guidetti G. Leavey G. (2015). Severe and enduring anorexia nervosa (SEED-AN): a qualitative study of patients with 20+ years of anorexia nervosa. Eur. Eat. Disord. Rev. 23, 318–326. 10.1002/erv.2367 26059633

[B55] Roncero M. Perpiña C. Marco J. H. Sanchez-Reales S. (2015). Confirmatory factoranalysis and psychometric properties of the Spanish version of the multidimensional body-self relations questionnaire-appearance scales. Body Image 14, 47–53. 10.1016/j.bodyim.2015.03.005 25867527

[B56] Rosenvinge J. H. Pettersen G. (2015a). Epidemiology of eating disorders part II: an update with a special reference to the DSM-5. Adv. Eat. Disord. 3, 198–220. 10.1080/21662630.2014.940549

[B57] Rosenvinge J. H. Pettersen G. (2015b). Epidemiology of eating disorders, part I: introduction to the series and a historical panorama. Adv. Eat. Disord. 3, 76–90. 10.1080/21662630.2014.898206

[B58] Sanz J. García-Vera M. P. Espinosa R. Fortun M. Vázquez C. (2005). Adaptación española del Inventario para la depresión de Beck-II (BDI-II): propiedades psicométricas en pacientes con trastornos psicológicos. Clín. Salud 16, 121–142.

[B59] Serpell L. Treasure J. Teasdale J. Sullivan V. (1999). Anorexia nervosa: friend or foe? Int. J. Eat. Disord. 25, 177–186. 10.1002/(SICI)1098-108X(199903)25:2<177::AID-EAT7>3.0.CO;2-D 10065395

[B60] Soler J. Vega D. Feliu-Soler A. Trujols J. Soto Á. Elices M.. (2013). Validation of the Spanish version of the borderline symptom list, short form (BSL-23). BMC Psychiatry 13, 139–146. 10.1186/1471-244X-13-139 23672691 PMC3658905

[B61] Stein K. F. Corte C. (2007). Identity impairment and the eating disorders: content and organization of the self-concept in women with anorexia nervosa and bulimia nervosa. Eur. Eat. Disord. Rev. 15, 58–69. 10.1002/erv.726 17676674

[B62] Stice E. Gau J. M. Rohde P. Shaw H. (2017). Risk factors that predict future onset of each DSM−5 eating disorder: predictive specificity in high-risk adolescent females. J. Abnorm. Psychol. 126, 38–51. 10.1037/abn0000219 27709979 PMC5215960

[B63] Stice E. Marti C. N. Durant S. (2011). Risk factors for onset of eating disorders: evidence of multiple risk pathways from an 8-year prospective study. Behav. Res. Ther. 49, 622–627. 10.1016/j.brat.2011.06.009 21764035 PMC4007152

[B64] Tomba E. Tecuta L. Schumann R. Ballardini D. (2017). Does psychological well-being change following treatment? An exploratory study on outpatients with eating disorders. Compr. Psychiatry 74, 61–69. 10.1016/j.comppsych.2017.01.001 28107643

[B65] Troop N. A. (2016). Social rank, rank-related life events and eating pathology. Eur. Eat Disord. Rev. 24, 75–77. 10.1002/erv.2386 26136369

[B66] Villarejo C. Fernández-Aranda F. Jiménez-Murcia S. Peñas-Lledó E. Granero R. Penelo E.. (2012). Lifetime obesity in patients with eating disorders: increasing prevalence, clinical and personality correlates. Eur. Eat. Disord. Rev. 20, 250–254. 10.1002/erv.2166 22383308 PMC3510304

[B67] Viñas F. Villar E. Caparrós B. Juan J. Cornellá M. Pérez I. (2004). Feelings of hopelessness in a Spanish university population: descriptive analysis and its relationship to adapting university, depressive symptomatology and suicidal ideation. Soc. Psychiatry Psychiatr. Epidemiol. 39, 326–334. 10.1007/s00127-004-0756-2 15085336

[B68] Vos J. Vitali D. (2018). The effects of psychological meaning-centered therapies on quality of life and psychological stress: a metaanalysis. Palliat. Support Care 16, 608–632. 10.1017/S1478951517000931 30246682

[B69] Weinberger N. A. Kersting A. Riedel-Heller S. G. Luck-Sikorski C. (2016). Body dissatisfaction in individuals with obesity compared to normal-weight individuals: a systematic review and meta-analysis. Obes. Facts 9, 424–441. 10.1159/000454837 28013298 PMC5644896

[B70] Westwood L. M. Kendal S. E. (2012). Adolescent client views towards the treatment of anorexia nervosa: a review of the literature. J. Psychiatr. Ment. Health Nurs. 19, 500–508. 10.1111/j.1365-2850.2011.01819.x 22070426

[B71] Wetzler S. Hackmann C. Peryer G. Clayman K. Friedman D. Saffran K.. (2020). A framework to conceptualize personal recovery from eating disorders: a systematic review and qualitative meta-synthesis of perspectives from individuals with lived experience. Int. J. Eat. Disord. 53, 1188–1203. 10.1002/eat.23260 32181532

[B72] Wildes J. E. Ringham R. M. Marcus M. D. (2010). Emotion avoidance in patients with anorexia nervosa: initial test of a functional model. Int. J. Eat. Disord. 43, 398–404. 10.1002/eat.20730 19670226 PMC2882494

