# Corrigendum: Meaning in Life Mediates Between Emotional Deregulation and Eating Disorders Psychopathology: A Research From the Meaning-Making Model of Eating Disorders

**DOI:** 10.3389/fpsyg.2022.849974

**Published:** 2022-02-15

**Authors:** Jose H. Marco, Montserrat Cañabate, Cristina Martinez, Rosa M. Baños, Verónica Guillen, Sandra Perez

**Affiliations:** ^1^Personality, Assessment and Psychological Treatment, University of Valencia, Valencia, Spain; ^2^University CEU Cardenal Herrera, Castellón de la Plana, Spain; ^3^Hospital Clínico Universitario de Valencia, Valencia, Spain; ^4^Personality, Assessment and Treatments, Catholic University of Valencia San Vicente Martyr, Valencia, Spain; ^5^CIBER Fisiopatología Obesidad y Nutrición (CIBEROBN), Madrid, Spain

**Keywords:** meaning in life, emotional deregulation, obesity, young women, eating disorders, meaning-making model

In the original article, we neglected to include a Funding statement. The correct Funding statement can be found below:

## Funding

Funding for the study was provided by (R + D + I) Projects of the State Programs Oriented to the Challenges of Society, within the framework of the State Research Plan Scientific and Technical and Innovation, with Code: PID2019-111036RB-I00, from Ministry of Science and Innovation of Spain.

The authors apologize for this error and state that this does not change the scientific conclusions of the article in any way. The original article has been updated.

## Publisher's Note

All claims expressed in this article are solely those of the authors and do not necessarily represent those of their affiliated organizations, or those of the publisher, the editors and the reviewers. Any product that may be evaluated in this article, or claim that may be made by its manufacturer, is not guaranteed or endorsed by the publisher.

